# Effects of sound source localization of masking sound on perception level of simulated tinnitus

**DOI:** 10.1038/s41598-022-05535-x

**Published:** 2022-01-27

**Authors:** Yamato Kubota, Kuniyuki Takahashi, Yoriko Nonomura, Tatsuya Yamagishi, Shinsuke Ohshima, Shuji Izumi, Yuka Morita, Naotaka Aizawa, Arata Horii

**Affiliations:** 1 Department of Otolaryngology Head Neck Surgery, Niigata University Graduate School of Medical and Dental Sciences, 1-757 Asahimachi-dori, Chuo-ku, Niigata, 951-8510 Japan; 2 Present Address: Department of Otolaryngology, Jichi Medical University Saitama Medical Center, 1-847 Amanuma, Omiya-ku, Saitama, 330-8503 Japan

**Keywords:** Diseases, Medical research

## Abstract

Tinnitus therapies have been combined with the use of varieties of sound/noise. For masking external sounds, location of the masker in space is important; however, effects of the spatial location of the masker on tinnitus are less understood. We aimed to test whether a masking sound location would affect the perception level of simulated tinnitus. The 4 kHz simulated tinnitus was induced in the right ear of healthy volunteers through an open-type earphone. White noise was presented to the right ear using a single-sided headphone or a speaker positioned on the right side at a distance of 1.8 m for masking the simulated tinnitus. In other sessions, monaurally recorded noise localized within the head (inside-head noise) or binaurally recorded noise localized outside the head (outside-head noise) was separately presented from a dual-sided headphone. The noise presented from a distant speaker and the outside-head noise masked the simulated tinnitus in 71.1% and 77.1% of measurements at a lower intensity compared to the noise beside the ear and the inside-head noise, respectively. In conclusion, spatial information regarding the masking noise may play a role in reducing the perception level of simulated tinnitus. Binaurally recorded sounds may be beneficial for an acoustic therapy of tinnitus.

## Introduction

Tinnitus is a common symptom, with an estimated morbidity rate Kindly check and of 5–15% in developed countries^1^ and affects the quality of life in 1–3% of the total population^2,3^. Recent advances have suggested that plasticity in the cerebral cortex accompanying hearing loss is responsible for tinnitus^4^. There has been an increase in tinnitus therapies based on hearing aids and tinnitus retraining therapy (TRT) using counselling methods and sound generators^5,6^ based on the neurophysiological model proposed by Jastreboff^7^. The latest guideline from the American Academy of Otolaryngology-Head and Neck Surgery Foundation (AAO-HNSF) for tinnitus recommends a hearing aid and cognitive behavioral therapy and with sound therapy as an option^8^.

TRT and numerous other tinnitus therapies have been combined with the use of sounds including white noise, music, and natural sounds^9^. These sounds may divert attention from tinnitus. However, they are usually delivered directly to the ears or head by earphones or headphones. For the masking of external sounds (not tinnitus), it is reported that the location of the masker in space is important^10^; however, the effects of the spatial location of the masking for true tinnitus are less understood. Recently, Searchfield et al.^11^ proposed the concept of spatial masking of tinnitus; the minimum masking level and desired level were achieved at a lower intensity level when using 3D maskers compared to 2D presentations of noise in tinnitus patients, suggesting the usefulness of spatial masking. Therefore, when the target sound and the masker sound are sufficiently closely overlapping, it is considered that the masking sound works by its direct masking effects on the target sound. On the other hand, when the target sound and the masking sound are separated from each other, the effect of diverting attention may have a more important role than the direct masking effect on the target sound.

**Figure 1:**
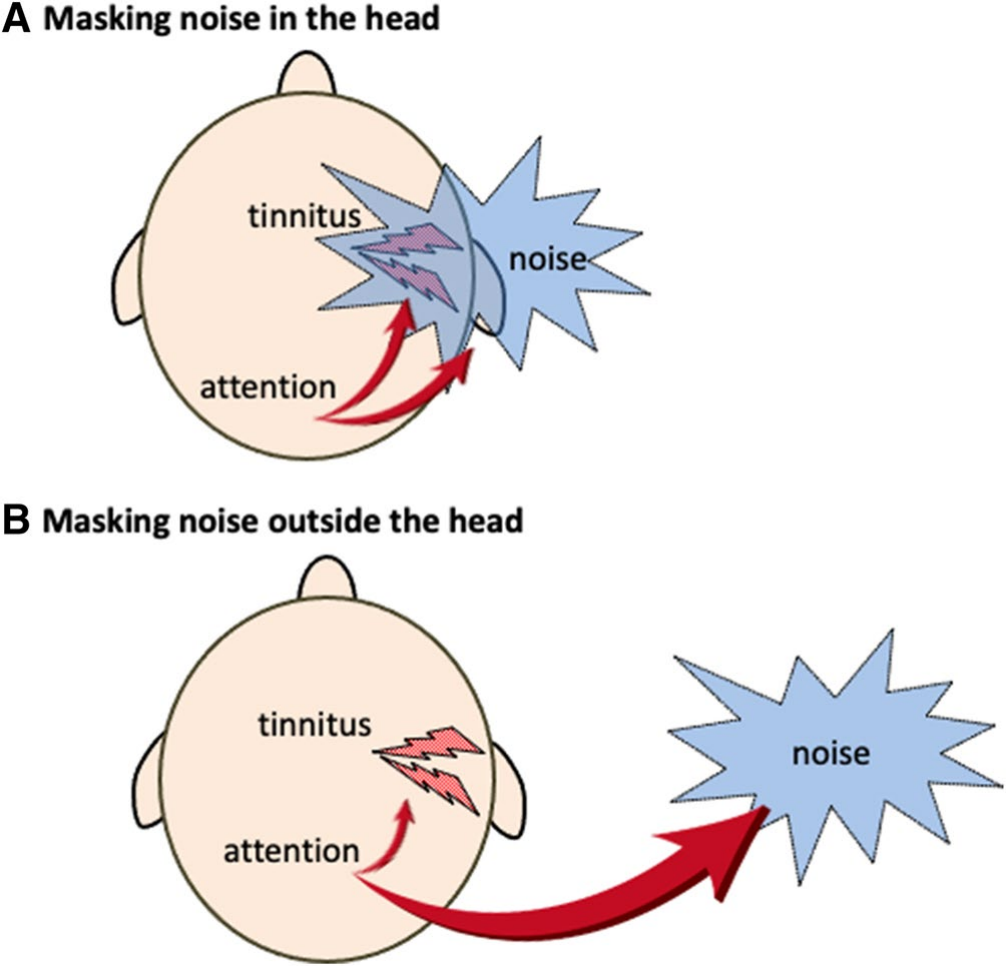
Working hypothesis on the relationship between attention and localization of masking noise. (**A**) Masking noise in the head. When tinnitus and the masking noise are at a close distance, the attention is focused on both. (**B**) Masking noise outside the head. The attention and perception of tinnitus may be reduced when the masking noise is at a distance from the tinnitus source. The blue splash means the masking noise, while the lightning shape means the simulated tinnitus.

Regarding the relationship between attention and tinnitus, Henry et al. reported the mitigating effect of music and environmental/conversational sounds by diverting attention from tinnitus^12^. Regarding the relationship between attention and listening ability, Allen et al. reported that speech perception thresholds rise when the direction of attention is different from the direction in which sounds are heard^12^. Therefore, since the sound source localization of tinnitus is inside the head close to the ear^14^, the treatment noise used in tinnitus therapy, if localized at a distance away from the head, may be beneficial in suppressing attention to tinnitus, thereby reducing the perception level of tinnitus (Fig. 1).

**Figure 2:**
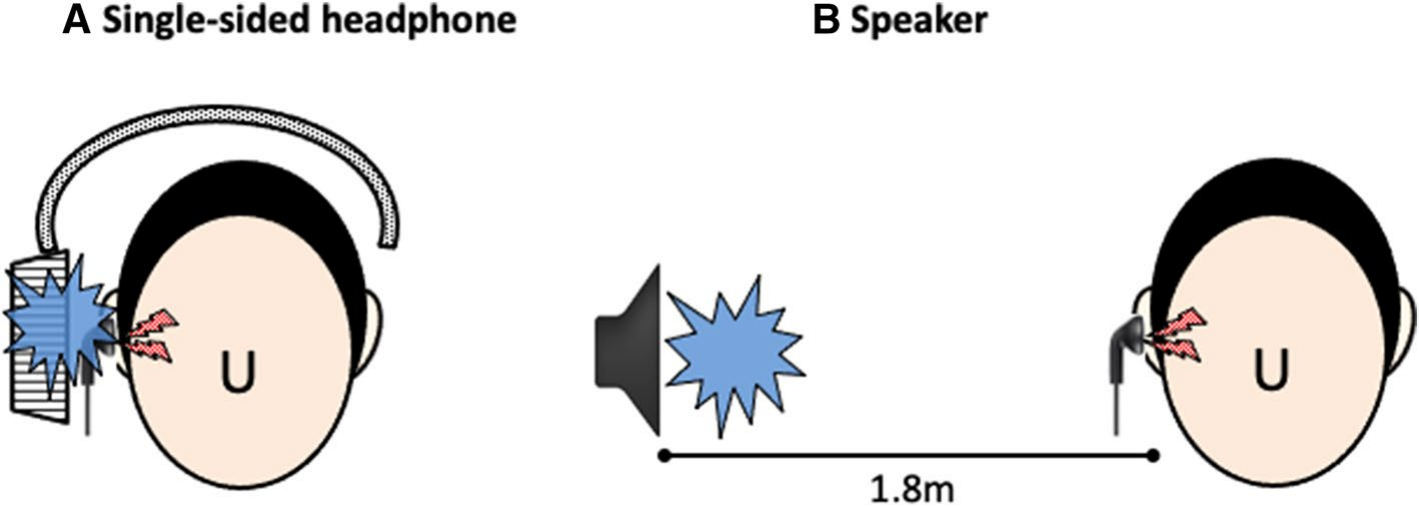
Schematic description of Experiment 1. The masking noise is presented (**A**) from a single-sided headphone or (**B**) from a speaker placed at a distance of 1.8 m away from the ear. The blue splash means the masking noise, while the lightning shape means the simulated tinnitus.

**Figure 3:**
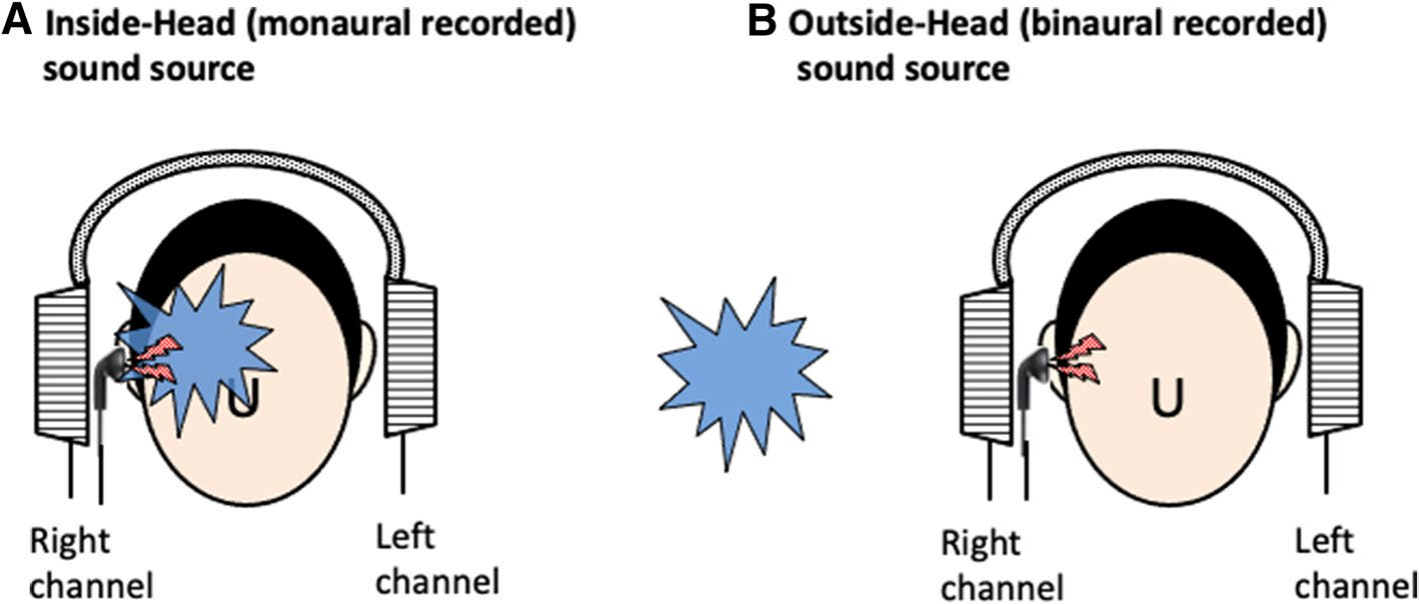
Schematic description of Experiment 2. (**A**) Inside-head (monaurally recorded) masking noise can be heard inside the head, whereas (**B**) Outside-head (binaurally recorded) masking noise can be heard outside the head. The blue splash means the masking noise, while the lightning shape means the simulated tinnitus.

In the present study, we attempted to verify the effects of spatial masking as well as of diverting attention on the perception level of simulated tinnitus. As a first step for utilizing in true tinnitus patients, we focused on simulated tinnitus induced in healthy volunteers, because there is large inter-individual variability in the sensation level required to mask true tinnitus. Simulated tinnitus was induced using an open-type earphone in one ear of each healthy volunteer. Then, comparisons in sound pressure required to sufficiently mask the simulated tinnitus were performed between masking noises presented from different sound source localizations (beside or distant from the head). This study consisted of two experiments. In Experiment 1, the masking noise was presented through a single-sided headphone (beside the head) or a speaker placed at 1.8 m lateral to the participant (distant from the head) (Fig. 2). In Experiment 2, monaurally or binaurally recorded noises presented to both ears through headphones, which were localized inside and outside the head, namely the inside-head and outside-head noise, respectively, were used as masking noises (Fig. 3). The left–right ratios of the sound pressure of the inside-head noise were adjusted to be the same as those of the outside-head noise. The sound pressure of noise to mask the simulated tinnitus was measured on a dummy head with a built-in microphone and sound level meter.

## Results

### Experiment 1

**Figure 4:**
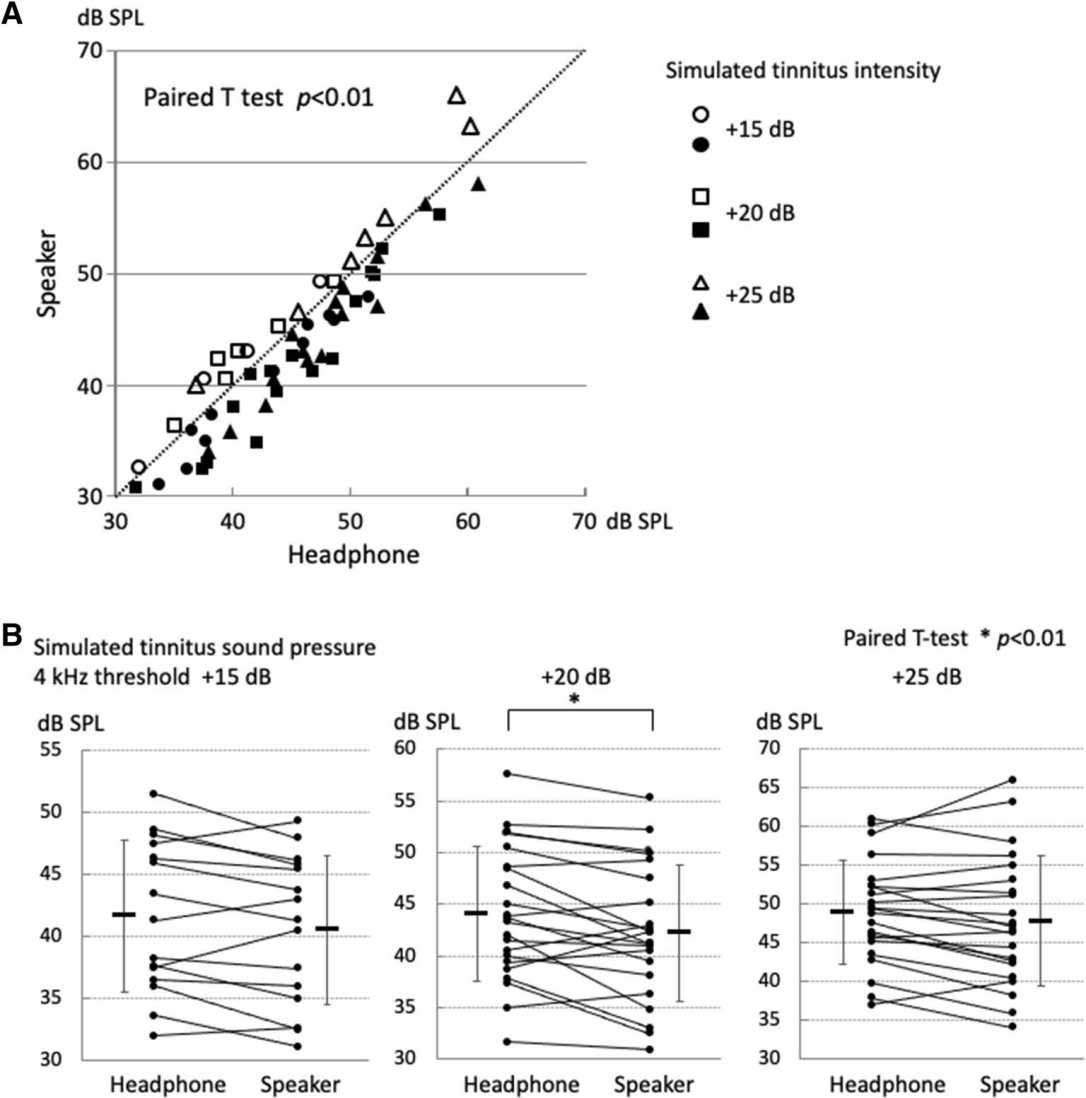
Masking effects on simulated tinnitus. (**A**) Sound pressure required to mask the simulated tinnitus in all inspections: headphone or speaker. In 71% (42/59) of measurements, simulated tinnitus could be masked at a lower sound pressure when the masking noise was delivered from the speaker (closed symbols). (**B**) Sound pressure required to mask simulated tinnitus of different intensities. Simulated tinnitus 20 dB above the hearing threshold was masked by the noise provided from the speaker at a significantly lower sound pressure level than the noise from the headphone. The horizontal bar next to the plot shows the mean, and the vertical error bars represent the standard deviation.

The 4 kHz hearing threshold of the participant’s right ear ranged from − 5 dB HL to 20 dB HL, and the average was 5.4 dB HL. The white noise emanating from the distant speaker masked the simulated tinnitus with smaller sound pressure than the white noise from the headphone for 71% (42/59 times) of the total threshold measurements (Fig. 4A, closed circle) (Paired *t* test, p < 0.01).

The masking sound pressure for each sound pressure of the simulated tinnitus was as follows:

1. threshold + 15 dB (n = 15): Headphone, mean 41.6 ± 5.93 dB SPL; Speaker, mean 40.5 ± 5.82 dB SPL (Paired *t* test, p = 0.057)
2. threshold + 20 dB (n = 22): Headphone, mean 44.0 ± 6.33 dB SPL; Speaker, mean 42.2 ± 6.42 dB SPL (Paired *t* test, p < 0.01)
3. threshold + 25 dB (n = 22): Headphone, mean 48.9 ± 6.53 dB SPL; Speaker, mean 47.8 ± 8.21 dB SPL (Paired *t* test, p = 0.116)

To test whether there is a difference in masking sound pressure due to two factors, the sound source position and the simulated tinnitus intensity, we tried to perform multiple comparison two-way ANOVA. As a result, there was no interaction between the sound source position and the simulated tinnitus intensity. Therefore, we performed the two-group comparison of the sound source position at three simulated tinnitus intensity levels with a paired *t* test with a Bonferroni correction (p < 0.05/3 = 0.016). When the sound pressure of the simulated tinnitus was set at the hearing threshold of + 20 dB, the white noise emanating from the distant speaker could mask the simulated tinnitus with significantly lower sound pressure than the white noise from the headphone (Paired *t* test, p < 0.01) (Fig. 4B). However, there were no significant differences in the masking effects between the headphone and the distant speaker among the three groups with different intensities of simulated tinnitus (Supplementary Fig. S1), justifying a paired *t* test with a Bonferroni correction.

**Figure 5:**
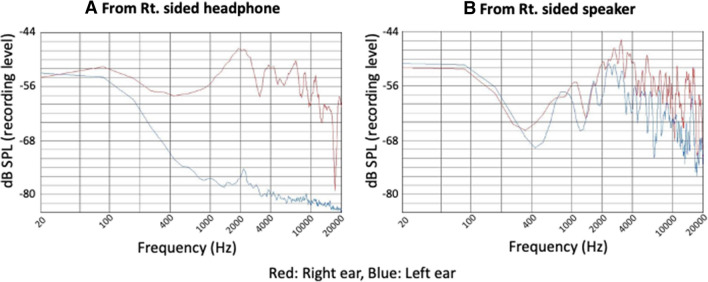
Sound spectrum of the masking noises from the single-sided headphone and the speaker. For the right ear, the sound intensity of noises from the speaker (**B**, red line) was weaker than that from the headphones (**A**, red line), at a frequency of 200 Hz to 2 kHz. However, the sound intensity at a frequency around 4 kHz, which is the frequency of the simulated tinnitus, from the right-sided speaker (**B**, red line), was almost the same as the noise from the right-sided headphone (**A**, red line). The sound intensity of around 4 kHz or higher was further attenuated in the left ear (**B**, blue line) than the right ear (**B**, red line) when the noise was delivered by the right-sided speaker.

Figure 5 shows the sound spectrum of the masking noises from the headphone (Fig. 5A) and the speaker (Fig. 5B). For the right ear, the sound intensity of noises from the speaker (Fig. 5B, red line) was weaker than that from the headphones (Fig. 5A, red line), at a frequency of 200 Hz to 2 kHz. However, the sound intensity at a frequency around 4 kHz, which is the frequency of the simulated tinnitus, from the right sided speaker (Fig. 5B, red line) was almost the same as the noise from the right sided headphone (Fig. 5A, red line). The sound intensity of around 4 kHz or higher was further attenuated in the left ear (Fig. 5B, blue line) than the right ear (Fig. 5B, red line) when the noise was delivered by the right sided speaker.

### Experiment 2

**Figure 6:**
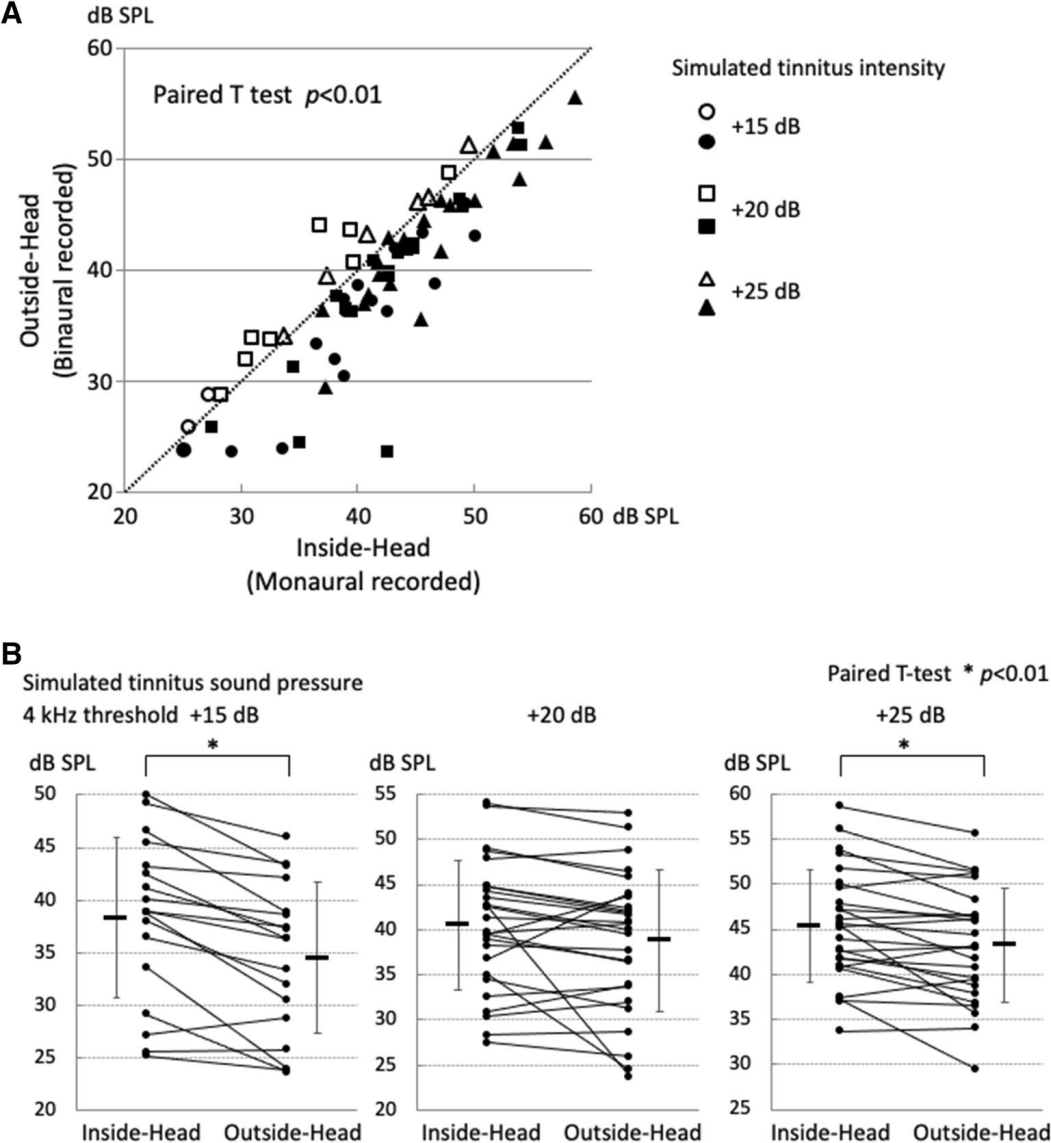
Masking effects on simulated tinnitus: outside-head vs. inside-head noises. (**A**) Sound pressure required to mask the simulated tinnitus in all inspections: outside-head vs. inside-head masking noises. In 77.1% (54/70) of measurements, simulated tinnitus could be masked with a lower sound pressure by the outside-head noise (closed symbols) compared to the inside-head noise (open symbols). (**B**) Sound pressure required to mask the simulated tinnitus of different intensities. All intensities of simulated tinnitus (+ 15, + 25 dB above the threshold) were masked by significantly lower sound pressures by the outside-head noise than the inside-head noise. The horizontal bar next to the plot shows the mean, and the vertical error bars represent the standard deviation.

The hearing threshold of the right ear at 4 kHz ranged from − 5 dB HL to 20 dB HL, with an average of 4.4 dB HL. In 54 of 70 threshold measurements (77.1%), simulated tinnitus could be masked with a lower sound pressure by the outside-head noise compared to the inside-head noise (Fig. 6A, closed circle) (Paired *t* test, p < 0.01).

The masking sound pressure for each sound pressure of the simulated tinnitus was as follows:

1. threshold + 15 dB (n = 18): Outside-Head, mean 34.5 ± 6.98 dB SPL; Inside-Head, mean 38.3 ± 7.43 dB SPL (Paired *t* test, p < 0.01)
2. threshold + 20 dB (n = 26): Outside-Head, mean 38.7 ± 7.68 dB SPL; Inside-Head, mean 40.4 ± 7.04 dB SPL (Paired *t* test, p = 0.074)
3. threshold + 25 dB (n = 26): Outside-Head, mean 43.3 ± 6.17 dB SPL; Inside-Head, mean 45.4 ± 6.08 dB SPL (Paired *t* test, p < 0.01).

Because there was no interaction in the two-way ANOVA as in Experiment 1, we performed a paired *t* test with a Bonferroni correction. Simulated tinnitus at the sound intensity of + 15 dB or + 25 dB of the 4 kHz threshold was masked by significantly lower sound pressures with the outside-head noise than the inside-head noise (Paired *t* test, p < 0.01) (Fig. 6B). However, there were no significant differences in the masking effects between the inside-head noise and the outside-head noise among the three groups with different intensities of the simulated tinnitus, justifying a use of one-way ANOVA test (Supplementary Fig. S2).

**Figure 7:**
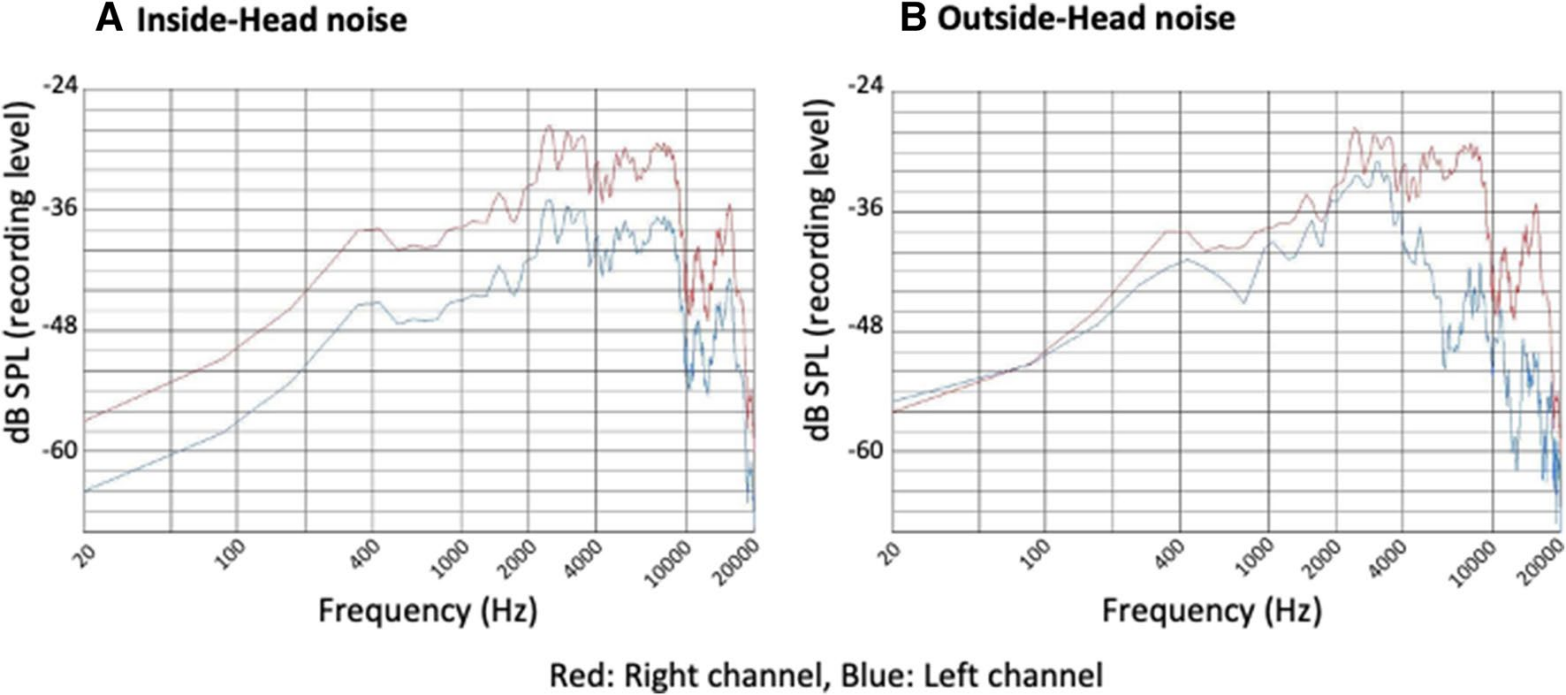
Sound spectrum of the inside-head (monaurally recorded) and outside-head (binaurally recorded) masking noise. (**A**) inside-head noise, (**B**) outside-head noise. For the right channel, the sound spectrums of the inside-head (monaurally recorded) noise (**A**, red line) and the outside-head (binaurally recorded) noise (**B**, red line) were identical. For the left channel, the sound spectrum of the inside-head noise (**A**, blue line) maintained the same shape as the right channel (**A**, red line), whereas the sound intensity decreased at 4 kHz or higher with the outside-head noise (**B**, blue line) compared with the right channel (**B**, red line).

As shown in Fig. 7, the sound spectrum of the inside-head noise (monaurally recorded) (Fig. 7A, red line) and the outside-head noise (binaurally recorded) (Fig. 7B, red line) measured for the right channel was identical. For the left channel, the sound intensity decreased at 4 kHz or higher with the outside-head noise (Fig. 7B, blue line) compared with right channel. In contrast, the sound spectrum of the inside-head noise for the left ear (Fig. 7A, blue line) maintained the same shape as the right channel but its intensity was dropped, since the sound pressure ratio of the left and right side had been adjusted to be equal to that of the outside-head noise.

### Subjective impressions of the masking noise

In 19 of 22 participants, masking noise from the distant speaker was significantly less unpleasant/uncomfortable than masking noise from the single-sided headphone (Wilcoxon signed rank test, p < 0.01). By comparison with the outside-head and inside-head noises, 24 of 26 participants responded that the outside-head noise was less unpleasant/uncomfortable (Wilcoxon signed rank test, p < 0.01).

## Discussion

In Experiment 1, in 71% of measurements, simulated tinnitus could be masked at a lower sound pressure when the masking noise was localized far from the ear (speaker) than when it was close to the ear (headphone) (Fig. 4A). A significant difference was observed when the simulated tinnitus was set at + 20 dB of the hearing threshold (Fig. 4B). Thus, sounds localized far from the ear are more effective in masking the simulated tinnitus. In Experiment 2, in 77.1% of measurements, simulated tinnitus could be masked at a lower sound pressure by the outside-head noise than the inside-head noise (Fig. 6A). For each sound pressure of simulated tinnitus (15 or 25 dB above the threshold), the outside-head noise could mask the simulated tinnitus at a significantly lower sound pressure than the inside-head noise (Fig. 6B). This observation is evidence of a higher masking effect on simulated tinnitus by sound sources localized outside the head.

It is well known that the masking effect is maximal when the target sound and the masking noise/sound arrive from the same direction or location. For instance, Kidd et al.^10^ conducted an experiment focusing on non-speech pattern-identification tasks in which the direction of the masking noise was varied from 0° to 180° on a horizontal plane. The results showed that distinguishing the target signal was strongly related to the spatial separation between the target signal and the masking noise: the closer in azimuth, the poorer the discrimination. Although many studies have shown the effect of spatial separation in azimuth, little is known about the effect of spatial separation in distance. Brungart and Simpson^15^ studied the effects of spatial separation in distance. Participants were asked to distinguish speech signals from speech noise, which were delivered by sources nearby at distances of 1 m, 25 cm, and 12 cm. It was shown that differences in distance play an important role in distinguishing signal from noise; the nearer the distance, the poorer the discrimination. In essence, these reports suggest that the masking effect may be higher when the masking sound is close to the target sound. However, the opposite result was obtained in the present study.

One possible explanation for this result is known as the “cocktail-party” effect, in which intelligibility improves when a target signal and an interfering sound source are spatially separated in azimuth^16,17^. In addition to azimuth, Westermann and Buchholz^18^ demonstrated that spatial separation in distance also has an important role in the perception of competing sound sources near the head and that informational masking sounds containing spatial information, such as direction and distance attained from binaural hearing, can help a subject efficiently perceive the target sound. In the present study, sound spectrum analysis of both the masking noise from the distant speaker in Experiment 1 and the outside-head noise in Experiment 2 revealed an attenuation of signal intensity at 4 kHz or higher on the left side compared to the right side (Figs. 5B, 7B), suggesting that these are the spatial information-containing informational-masking sounds. By contrast, the sound spectrum of the left channel of the inside-head noise (Fig. 7A, blue line) had the same shape as that of the right channel (Fig. 7A, red line), lacking the spatial information. Not only differences in this sound spectrum but also in phase would provide spatial information such as distance and direction to the masking noise from the distant speaker and the outside-head noise. This would account for the higher masking effect of noise from the speaker and the outside-head noise than that from the single-sided headphone and the inside-head noise, respectively. Comparing masking noise from the speaker and the outside-head noise, the outside-head noise seemed to have better masking effects than the noise from the speaker (see, Figs. 4B and 6B). This is probably because the masking sound from the distant speaker may not provide sufficient signals to construct spatial information compared to the outside-head noise, when it reaches the ear as revealed by the sound spectrum analysis (Figs. 5, 7). Since the experiments were performed in a narrow sound shielded room, sound reflection of the high-tone components from the wall may have also reached the contralateral ear, resulting in a decrease in the right–left differences in masking sounds when provided by the speaker (Fig. 5B).

Another explanation for this result is that increased attention to the masking noise plays an important role in the perception level of the simulated tinnitus. Using a speech signal and masker, Allen et al.^13^ presented sounds at 20°, 40°, and 60° from the front-facing direction of the participants. A speech signal was presented from either the direction from which the participants were expecting it (attended) or the direction from which they were not (unattended). The authors reported that speech perception thresholds were increased from angles at which the speech signal was unexpected and that the perception level was reduced when the target sound and attention were separated spatially. In addition to the effects of angle differences between the target sound and attention on sound perception^13^, the present results suggest that distance between the target sound (simulated tinnitus) and attention (masking noise) may affect the target sound perception level. The perception level of simulated tinnitus can be suppressed by dispersing the attention to the sound source of simulated tinnitus localized within the head through attention to a masking noise located outside the head. That is, when the target sound and the masking noise are separated from each other, the effect of dispersing attention may have a more important role than the “masking” effect. For further dispersing attention, there is a possibility to give better effects using a masking noise that emulates the sound moving outside the head three-dimensionally.

Subjective impressions of the masking noise showed that the localization of the sound source outside the head (distant speaker or the outside-head noise) led to significantly less unpleasantness and discomfort for the subject than when the sound source was localized in the near field (beside the ear or the inside-head noise). Reducing the unpleasant and uncomfortable feelings are the major issues relating to the continued use of the acoustic treatment devices in tinnitus patients. Given that the reduction of perception level of tinnitus by masking noise outside the head was significant but small (Figs. 4B, 6B), these results suggest that the binaurally recorded noise localized externally to the head could reduce the challenges faced when using medical devices to treat tinnitus by reducing the discomfort associated with treatment sounds. In addition, Searchfield et al.^11^ reported that a slightly better outcome was obtained after using 3D maskers than TRT using a conventional masker when evaluated by the Tinnitus Functional Index. Binaurally recorded comfortable sounds localized outside the head may be worthy of trial as a sound for acoustic therapy for tinnitus patients.

A limitation of the study is that simulated tinnitus used in this study is known to behave differently from true tinnitus. For instance, patients with noise-induced tinnitus have prolonged reaction time and event related potentials (ERPs) that are reduced in amplitude and prolonged in latency^19^; however, simulated tinnitus applied by a standard audiometric vibrator could not affect the ERPs when presented to patients with noise-induced hearing loss without tinnitus^20^. It should be understood that simulated tinnitus is different from true tinnitus^21^. Moreover, we used simulated tinnitus at a relatively high sound level such as + 15 to + 25 dB above the hearing threshold^22^, since some participants reported that the simulated tinnitus was too mild to recognize. Indeed, true tinnitus is usually perceived only a few decibels above the hearing threshold^23^. Another limitation is that the sound pressure level of the masking sounds was measured in the ear canal of a dummy head but not in the subject with the real ear measurement (REM). When comparing the speaker with the headphone, the loudness would be different even when both sound pressure levels are adjusted to the same, because the waveform delivered on the eardrum would change due to the room acoustics and the interaural coherence^24^. In Experiment 2, there is no room acoustics because both masking sounds are generated from headphones, but the individual ear canal shape may affect the waveform and the loudness. Therefore, if the sound pressure level of the masking sounds is measured by REM, the experiments can become more precise.

## Methods

### Ethics statement

This study was approved by the IRB of Niigata University Hospital (approval #2017-0232). Each participant provided informed consent to take part in the experiments. We assert that all procedures contributing to this work comply with the ethical standards of the relevant national and institutional guidelines on human experimentation and with the Helsinki Declaration of 1975, as revised in 2008.

### Experiment settings

The experiment was conducted in the sound shielded room (AT-71, RION Co., Ltd. Japan: W1830 mm × D1830 mm × H1925 mm). The participants were volunteers with no aural disorder. The participants undertook pure tone audiometry testing prior to the experiments to evaluate their hearing level, as they were healthy volunteers without subjective tinnitus. All participants had a normal hearing level in both ears that was within 20 dB at 125 Hz, 250 Hz, 500 Hz, 1 kHz, 2 kHz, 4 kHz, and 8 kHz. Two experiments were subsequently conducted in which an open-type earphone (SONY MDR-888), which keeps ears open to catch ambient sounds and generates no occlusion effects of external ear canals, was used to deliver a simulated tinnitus sound to one ear. In Experiment 1, the masking noise was presented from the same side using a single-sided headphone (STAX SR-L500) or a speaker (YAMAHA NS-10MMT) positioned on the same side at a distance of 1.8 m. In Experiment 2, two types of masking noise with different sound localizations were presented from a dual-sided headphone (STAX SR-L500); one was localized inside the head (monaurally recorded noise) and the other was localized outside the head (binaurally recorded noise).

### Experiment 1

Experiment 1 included 22 participants (19 men and 3 women), aged between 19 and 43 years (average, 29.2 years).

#### Simulated tinnitus sound

The simulated tinnitus sound consisted of a pure tone at 4 kHz that was presented in the right ear of the participant using an open-type earphone (SONY MDR-888). The 4 kHz tone was generated by the function generator (TEXIO FG-274) and amplified with an analogue amplifier (FOSTEX HP-A7), which was adjusted to + 15 dB, + 20 dB, or + 25 dB of the participant’s 4 kHz hearing threshold for the right ear with an attenuator. Seven participants who had a low hearing threshold of 4 kHz so that + 15 dB simulated tinnitus was too small to be sensed under the environmental noise in the soundproof room were excluded from the + 15 dB experiment.

#### Masking sound

The simulated tinnitus was masked by white noise delivered to the right ear through open-type headphones (STAX SR-L500/driven by STAX SRM-353X analogue headphone amplifier) (Fig. 2A) or by white noise presented by a speaker (YAMAHA NS-10MMT/driven by LUXMAN M-200 analogue amplifier) installed 1.8 m lateral to the right ear of the participant (Fig. 2B). The order of presentation of the white noise emitted from the headphone or speaker was randomized for each subject.

#### Measurement of the masking threshold

During simulated tinnitus, the sound pressure of the white noise from the headphone or the distant speaker was increased, until sufficient masking of the simulated tinnitus was obtained. Subsequently, the sound pressure of the white noise was gradually decreased, and the threshold at which the participants became aware of the simulated tinnitus was stipulated as the masking sound pressure threshold.

The sound pressure at that point was measured using a dummy head that had a built-in microphone (SOUTHERN ACOUSTICS Co., Ltd, Japan. Type 3500 with Type 2128E microphone, ACO Co., Ltd) installed under the same conditions as those used with the participants. A sound level meter (NA-42S RION CO., Ltd, Japan) was also used in every trial.

#### Sound spectrum analysis of the masking noise

The sound spectrum of noises from the single-sided headphone and speaker were analyzed. Two noises at an intensity of 45 dB SPL in the right ear were recorded in both sides using a dummy head microphone placed in identical conditions to those that were used with the participants. The sound spectrum was analyzed using Sony Sound Forge Pro 11.0. Forty-five dB SPL was chosen since the measurement of the masking threshold revealed that this intensity was close to the mean intensity of the masking noises used in all assessments.

### Experiment 2

In Experiment 1, the masking noise delivered by a speaker reached the contralateral ear as well as the ipsilateral ear to which the simulated tinnitus was applied. Thus, we cannot dismiss a masking effect on both ears. In Experiment 2, the masking noise was delivered to both ears by the same headphones but with different localizations of the sound source; inside-head (monaurally recorded) noise vs. outside-head (binaurally recorded) noise. Experiment 2 included 26 participants (22 men and 4 women) aged between 19 and 44 years (average, 28.8 years).

#### Simulated tinnitus sound

As in Experiment 1, a 4 kHz pure tone 15 dB, 20 dB, or 25 dB above the participant’s 4 kHz hearing threshold was presented to the participant’s right ear through an open-type earphone (SONY MDR-888). Seven participants who had a low hearing threshold of 4 kHz so that + 15 dB simulated tinnitus was too small to be sensed under the environmental noise in the soundproof room were excluded from the + 15 dB experiment.

#### Masking sound

Binaural recording is a type of recording method where microphones are built into both ears of a dummy head, which resembles a human head. By reproducing the binaurally recorded sound with headphones, outside-head localization with realism is obtained^25^. In contrast, when a monaurally recorded sound is reproduced to both ears with headphones, the sound from the headphones is projected onto the ear axis (the straight line connecting the entrances of the ear canal of the left and right ears), which is localized within the inside-head sound field^26^.

Two types of noise, namely inside-head and outside-head noise, which were monoaurally and binaurally recorded sound sources, were prepared beforehand and presented to both ears through open-type headphones (STAX SR-L500). When recording the binaural sound, a dummy head with bilateral built-in microphones was placed at the center of a sound proof room (W 4000 mm × D 4000 mm × H 2350 mm). The white noise emitted from the speaker (YAMAHA NS-10 MMT/driven by LUXMAN M-200 analogue amplifier) placed 3 m, 90 degrees from the right ear was recorded with an IC recorder (SONY ICD-SX2000: linear PCM 44.1 kHz/16 bit) connected with bilateral built-in microphones. When listening the inside-head noise with headphones, all participants perceived it inside the head on the right side (Fig. 3A). When the outside-head noise was presented through headphones, all participants perceived the noise at the outside of their heads on the right side, but they all could not perceive it uniformly at 3 m; some felt a little near and others distant (Fig. 3B). For the preparation of the inside-head noise, the component of the right channel of the aforementioned outside-head noise was presented to both ears. The sound pressure ratio of the left and right side was adjusted to be equal to that of the left and right ratio of the outside-head noise. The order of presentation of the inside-head or outside-head noise was randomized for each subject. Then, the sound spectrum of these two noises recorded in the right and left channels was analyzed by the same methods as described in Experiment 1.

#### Measurement of the masking threshold

The method for measuring the masking sound pressure was the same as the method used in Experiment 1.

#### Subjective impressions of the masking noise

At the end of the study trials, the participants were asked “which of the masking noises used would be the least unpleasant and present minimal discomfort if listened to for a long period of time?”: the single-sided headphones vs. the speaker, and the inside-head noise vs. the outside-head noise.

### Statistics

Paired *t* test was used to compare repeated measurements and Wilcoxon signed rank test was used to analyze for subjective impressions. All statistical tests were performed with SPSS ver. 21. The significance level was set at p < 0.05.

## Supplementary Information

Supplementary Figure S1.

Supplementary Figure S2.

Supplementary Legends.

## References

[1] Eggermont JJ, Roberts LE (2004). The neuroscience of tinnitus. Trends Neurosci..

[2] Fujii K (2011). Prevalence of tinnitus in community-dwelling Japanese adults. J. Epidemiol..

[3] Kim HJ (2015). Analysis of the prevalence and associated risk factors of tinnitus in adults. PLoS One.

[4] Eggermont JJ (2007). Pathophysiology of tinnitus. Prog. Brain Res..

[5] Jastreboff PJ, Hazell JW (1993). A neurophysiological approach to tinnitus: Clinical implications. Br. J. Audiol..

[6] Jastreboff PJ, Gray WC, Gold SL (1996). Neurophysiological approach to tinnitus patients. Am. J. Otol..

[7] Jastreboff PJ (1990). Phantom auditory perception (tinnitus): Mechanisms of generation and perception. Neurosci. Res..

[8] Tunkel DE (2014). Clinical practice guideline: Tinnitus. Otolaryngol. Head Neck Surg..

[9] Henry JA, Rheinsburg B, Zaugg T (2004). Comparison of custom sounds for achieving tinnitus relief. J. Am. Acad. Audiol..

[10] Kidd G, Mason CR, Rohtla TL, Deliwala PS (1998). Release from masking due to spatial separation of sources in the identification of nonspeech auditory patterns. J. Acoust. Soc. Am..

[11] Searchfield GD (2016). Spatial masking: Development and testing of a new tinnitus assistive technology. Assist. Technol..

[12] Henry JA, Zaugg TL, Myers PJ, Schechter MA (2008). Using therapeutic sound with progressive audiologic tinnitus management. Trends Amplif..

[13] Allen K, Alais D, Carlile S (2009). Speech intelligibility reduces over distance from an attended location: Evidence for an auditory spatial gradient of attention. Atten. Percept. Psychophys..

[14] Searchfield GD, Kobayashi K, Proudfoot K, Tevoitdale H, Irving S (2015). The development and test–retest reliability of a method for matching perceived location of tinnitus. J. Neurosci. Methods.

[15] Brungart DS, Simpson BD (2002). The effects of spatial separation in distance on the informational and energetic masking of a nearby speech signal. J. Acoust. Soc. Am..

[16] Ericson M, McKinley R, Gilkey RH, Anderson TR (2001). Binaural and Spatial Hearing in Real and Virtual Environments.

[17] Bronkhorst AW (2000). The cocktail party phenomenon: A review of research on speech intelligibility in multiple-talker conditions. Acta Acust. Acust..

[18] Westermann A, Buchholz JM (2015). The effect of spatial separation in distance on the intelligibility of speech in rooms. J. Acoust. Soc. Am..

[19] Attias J, Urbach D, Gold S, Shemesh Z (1993). Auditory event related potentials in chronic tinnitus patients with noise induced hearing loss. Hear. Res..

[20] Attias J, Bresloff I, Furman V, Urbach D (1995). Auditory event related potentials in simulated tinnitus. J. Basic Clin. Physiol. Pharmacol..

[21] Penner MJ (1993). Synthesizing tinnitus from sine waves. J. Speech Hear. Res..

[22] Seimetz BM (2016). Pitch and loudness tinnitus in individuals with presbycusis. Int. Arch. Otorhinolaryngol..

[23] Basile C, Fournier P, Hutchins S, Hébert S (2013). Psychoacoustic assessment to improve tinnitus diagnosis. PLoS One.

[24] Denk F, Kohnen M, Llorca-Bofí J, Vorländer M, Kollmeier B (2021). The, "Missing 6 dB" revisited: Influence of room acoustics and binaural parameters on the loudness mismatch between headphones and loudspeakers. Front. Psychol..

[25] Møller H (1992). Binaural technology—Fundamentals. J. Appl. Acoust..

[26] Blauert J (1996). Spatial Hearing: The Psychophysics of Human Sound Localization.

