# Supplementary figures and images for: Effects of sound source localization of masking sound on perception level of simulated tinnitus
Source: Sci Rep. 2022 Jan 27;12:1452. doi: 10.1038/s41598-022-05535-x (PMC8795453; doi:10.1038/s41598-022-05535-x)

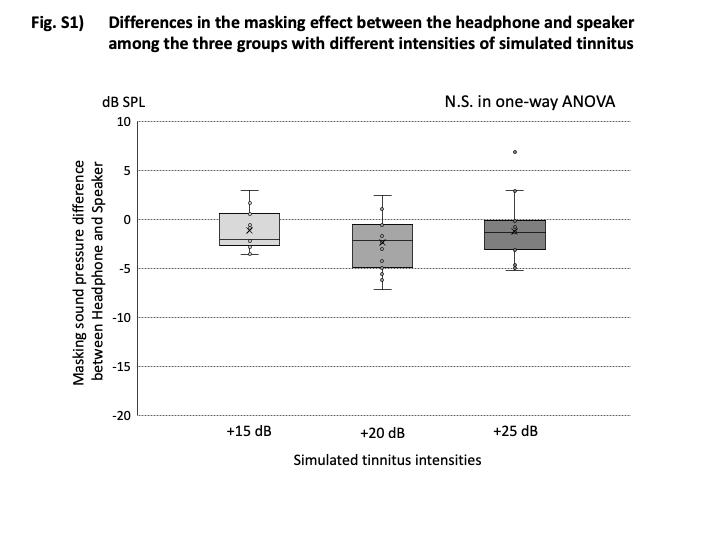

Supplement: Supplementary file 1 — Supplementary Figure S1. [file 41598_2022_5535_MOESM1_ESM.tiff]

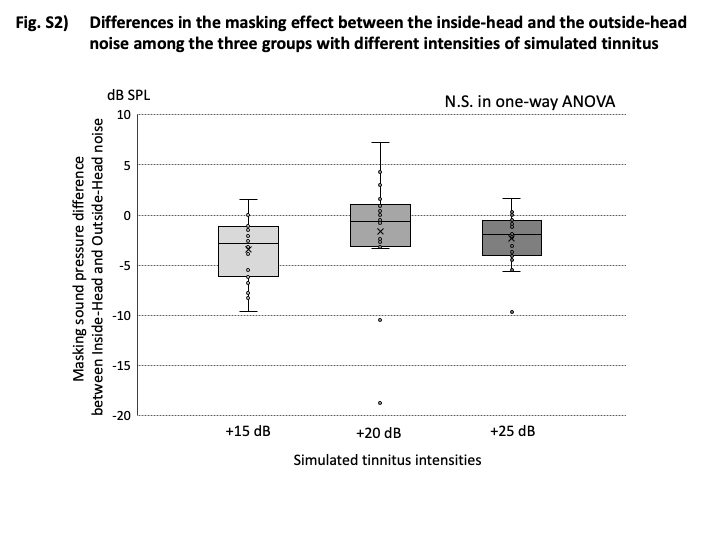

Supplement: Supplementary file 2 — Supplementary Figure S2. [file 41598_2022_5535_MOESM2_ESM.tiff]
