# Supplementary material for: Effects of sound source localization of masking sound on perception level of simulated tinnitus
Source: Sci Rep. 2022 Jan 27;12:1452. doi: 10.1038/s41598-022-05535-x (PMC8795453; doi:10.1038/s41598-022-05535-x)
Supplement: Supplementary file 3 — Supplementary Legends. [file 41598_2022_5535_MOESM3_ESM.docx]

Fig. S1: Differences in the masking effect between the headphone and speaker among the three groups with different intensities of simulated tinnitus.

One-way ANOVA showed no statistically significant difference in the masking effects among three different intensities.

Fig. S2: Differences in the masking effect between the inside-head and the outside-head noise among the three groups with different intensities of simulated tinnitus.

One-way ANOVA showed no statistically significant difference in the masking effects among three different intensities.
